# Estimating genome-wide regulatory activity from multi-omics data sets using mathematical optimization

**DOI:** 10.1186/s12918-017-0419-z

**Published:** 2017-03-27

**Authors:** Saskia Trescher, Jannes Münchmeyer, Ulf Leser

**Affiliations:** Knowledge Management in Bioinformatics, Computer Science Department, Humboldt-Universität zu Berlin, Unter den Linden 6, 10099 Berlin, Germany

**Keywords:** Gene regulation, Regulatory network, Systems biology, Mathematical optimization

## Abstract

**Background:**

Gene regulation is one of the most important cellular processes, indispensable for the adaptability of organisms and closely interlinked with several classes of pathogenesis and their progression. Elucidation of regulatory mechanisms can be approached by a multitude of experimental methods, yet integration of the resulting heterogeneous, large, and noisy data sets into comprehensive and tissue or disease-specific cellular models requires rigorous computational methods. Recently, several algorithms have been proposed which model genome-wide gene regulation as sets of (linear) equations over the activity and relationships of transcription factors, genes and other factors. Subsequent optimization finds those parameters that minimize the divergence of predicted and measured expression intensities. In various settings, these methods produced promising results in terms of estimating transcription factor activity and identifying key biomarkers for specific phenotypes. However, despite their common root in mathematical optimization, they vastly differ in the types of experimental data being integrated, the background knowledge necessary for their application, the granularity of their regulatory model, the concrete paradigm used for solving the optimization problem and the data sets used for evaluation.

**Results:**

Here, we review five recent methods of this class in detail and compare them with respect to several key properties. Furthermore, we quantitatively compare the results of four of the presented methods based on publicly available data sets.

**Conclusions:**

The results show that all methods seem to find biologically relevant information. However, we also observe that the mutual result overlaps are very low, which contradicts biological intuition. Our aim is to raise further awareness of the power of these methods, yet also to identify common shortcomings and necessary extensions enabling focused research on the critical points.

**Electronic supplementary material:**

The online version of this article (doi:10.1186/s12918-017-0419-z) contains supplementary material, which is available to authorized users.

## Background

**Fig. 1:**
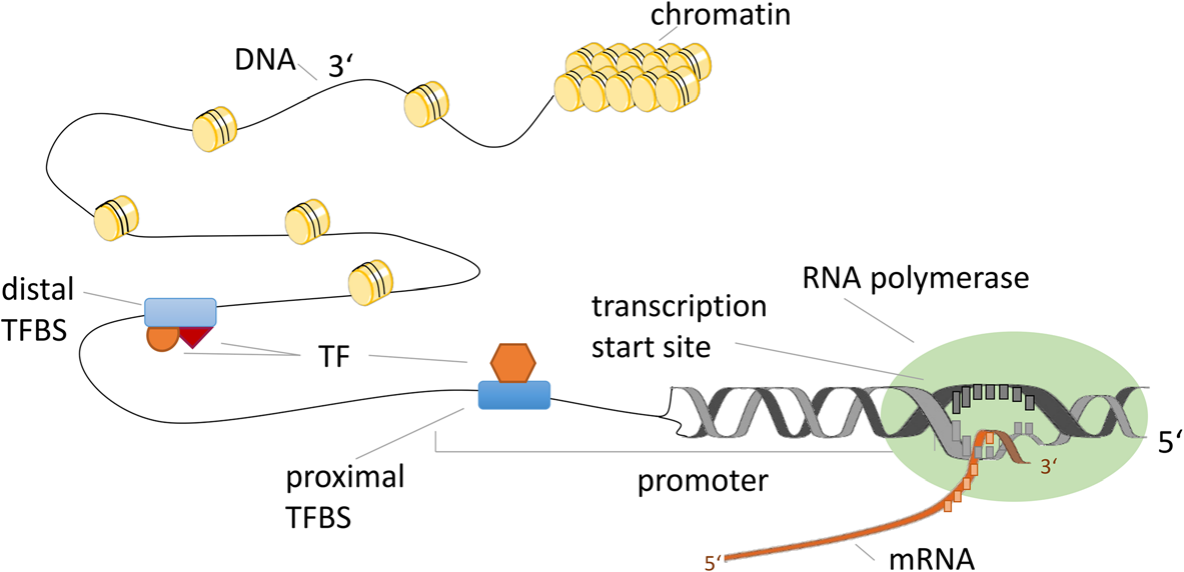
Transcription of DNA into RNA. Transcription factors (TFs) bind to distal or proximal TF binding sites (TFBS) enhancing the binding of RNA polymerase and activating the transcription of DNA into RNA

Gene regulation is one of the most important biological processes in living cells. It is indispensable for adapting to changing environments, stimuli, and developmental stage and plays an essential role in the pathogenesis and course of diseases. Mechanistically, the transcription of DNA into RNA is predominantly controlled by a complex network of transcription factors (TFs) (see Fig. 1). These proteins bind to enhancer or promoter regions adjacent to the genes they regulate [1], which may enhance or inhibit the recruitment of RNA polymerase and thereby activate or repress gene transcription [2]. Gene products also can be modified post-translationally via microRNAs (miRNAs) degrading the transcript or inhibiting their translation [3]. Besides, a multitude of other mechanisms influence gene regulation, such as chromatin remodelling [4], epigenetic effects [5], and compound-building of transcription factors [2]. Distortion of regulatory processes is inflicted with various diseases [6, 7], especially with cancer [8, 9].

Due to this importance, many efforts have been devoted to the elucidation of human regulatory relationships and networks. Wide-spread experimental techniques are transcriptome measurements to quantify gene and transcription factor co-expression [10], chromatin immunoprecipitation (ChIP) on chips or followed by sequencing for identifying binding patterns of specific TFs [11], and bisulfite sequencing to find epigenetic signals of regulation [12]. Many large-scale datasets of such experiments have been published and are available in public repositories such as the Gene Expression Omnibus (GEO) [13], the Cancer Genome Atlas (TCGA) [14] or the Encyclopedia of DNA Elements (ENCODE) [15]. Computational methods are also used, for instance, to identify transcription factor binding sites (TFBS) [16] or to find known TFBS within the genome (e.g., [17, 18]). Several databases have been created which store relevant information, such as lists of binding motifs (TRANSFAC [19] or JASPAR [20]) or targets of regulatory miRNAs [21].

Such measurements and predictions are used by network reconstruction algorithms to predict regulatory relationships and regulatory networks [22]. A plethora of different methods have been proposed, ranging from purely qualitative methods [23] over simple statistical approaches [24] to more advanced probabilistic frameworks [25]. Early methods were plagued by insufficient data and a general scarcity of background knowledge, which led to rather unstable results [26]. This situation has changed dramatically over the last years, as results of more and more large screens have been made publicly available [27] and also the knowledge on principal regulatory relationships has increased [28, 29]. This, in turn, has increased the interest in methods which predict genome-wide networks using a systematic, unified, mathematical framework.

Here, we review five rather recent methods and conduct a quantitative comparison of their results with the goal to identify their mutual strengths and weaknesses. They all have in common that they assume both the set of regulators (transcription factors or micro RNAs) to be known and the topology of the regulatory network to be given. By combining this background knowledge with specific omics data sets, especially transcriptome data, they try to infer the activity of regulators in a certain experimental condition or disease using mathematical optimization. All presented methods are global methods in the sense that they compute activities genome-wide (as much as represented by the underlying network), thus removing the shortcomings of local methods which ignore cross-talk between sub-models and global effects within samples. The methods predominantly produce a ranked list of regulators, sorted by their activity in a given group of samples; given that a multitude of biological influences is ignored during inference, especially kinetic and temporal effects, their goal cannot be to produce absolute snapshots of regulatory activity. We describe each method in detail and compare them with respect to the most important properties, such as the data being used, the method applied for deriving optimized activity values, or the evaluation performed to show effectiveness. We further implemented a quantitative comparison including four of the presented methods to objectively analyze their results. As contrast, we also include ARACNE [30] as sixth method; this algorithm uses only local reasoning and requires no background knowledge, but is still rather popular.

## Methods

We describe in detail five methods which infer transcription factor activity from omics data sets using a background network of transcription factors and the genes they regulate. All use some form of mathematical optimization. To emphasize the common ground of these at-first-sight rather different methods, we explain their underlying models using a simple framework for defining the relationships of transcription factors and genes. This framework is presented first; it should be understood as a least common denominator, not as a proper method for network inference by itself. We then describe five recently published methods for genome-wide TF activity estimation as extensions or constraints to this general framework, namely the approach by Schacht et al. [31] (estimation of TF activity by the effect on their target genes), RACER [32], RABIT [33], ISMARA [34] and biRte [35]. Additionally, we contrast these more comprehensive methods with the local inference algorithm ARACNE [30], a popular tool for the de-novo reconstruction of gene regulatory networks. Key properties of all methods (input, mathematical model, computation, output) are summarized in Table 1.

**Table 1:** Overview of methods for estimating regulatory activity from transcriptome data comparing input data, modelling, computational aspects and outcome variables

Method	Input	Model	Computation	Output
Approach by Schacht et al.	- mRNA expression data - TF binding information	Linear model $\widehat{g_{i, s}}= c+{\displaystyle \sum_t}{\beta}_t{b}_{t, i}\left({\theta}_{a, t} ac{t}_{t, s}+{\theta}_{g, t}{g}_{t, s}\right)$ with $a c{t}_{t, s}=\frac{{\displaystyle {\sum}_i}{b}_{t, i}{g}_{i, s}}{{\displaystyle {\sum}_i}{b}_{t, i}},\ {\theta}_{a, t}+{\theta}_{g, t}=1,{\theta}_{a, t},{\theta}_{g, t}\in \left\{0,1\right\}$	- Optimization criterion: minimize sum of absolute errors - Mixed-integer linear programming - Optimization via Gurobi 5.5	- parameter for each TF: *β*_*t*_ - decision for each TF if *θ*_*a*,*t*_or *θ*_*g*,*t*_ was chosen
RACER	- mRNA expression data - copy number variation - DNA methylation - miRNA expression signals - TF binding information - miRNA target site info (*c*)	Linear models: 1) $\widehat{g_{i, s}}= c+{\theta}_{CNV, s} C N{V}_{i, s}+{\theta}_{DM, s} D{M}_{i, s}+{\displaystyle \sum_{t\ }}{\beta}_{t, s}\ {b}_{t, i} + {\displaystyle \sum_{mi\ }}{\beta}_{mi, s}\ {c}_{i, mi} miRN{A}_{mi, s}$ 2) $\widehat{g_{i, s}}=\tilde{c}+{\tilde{\theta}}_{i, CNV} C N{V}_{i, s}+{\tilde{\theta}}_{i, DM} D{M}_{i, s}+{\displaystyle \sum_{t\ }}{\gamma}_{i, t}\ {\beta}_{t, s} + {\displaystyle \sum_{mi\ }}{\gamma}_{i, mi}\ {\beta}_{mi,\mathrm{s}}$	- Optimization criterion: minimize sum of squared errors with L_1_ norm penalty on linear coefficients - Elastic-net regularized generalized linear models and LASSO	1) sample-specific TF and miRNA activities *β*_*t*,*s*_ and *β*_*mi*,*s*_ 2) TF-gene *γ*_*i*,*t*_ and miRNA-gene *γ*_*i*,*mi*_interactions across all samples
RABIT	- differential mRNA expression data - somatic mutations - DNA methylation - copy number variation - TF binding info - recognition motifs for RNA-binding protein (RBP)	Linear model: $\widehat{g_t} = {\displaystyle \sum_f}{\theta}_f{B}_{f, i} + {\displaystyle \sum_t}{\beta}_t{b}_{t, i}$ With *B*: background factors (gene CNA, promoter DNA methylation, promoter degree promoter CpG content)	- Frisch-Waugh-Lovell method, select subset of significant TFs via model selection procedure and remove TFs with insignificant correlation across tumors	- regulatory activity score for each TF (t value of linear regression coefficient of *t*-test)
ISMARA	- gene expression or chromatin state measurements - annotation of promoters (number of predicted sites for motifs) - transcripts and associated promoters - miRNA target site predictions	Linear model $\widehat{g_{p, s}}={c}_p+{c}_s+{\displaystyle \sum_m}{N}_{p, m}\ {\beta}_{m, s}$	- Optimization criterion: minimize sum of errors - Bayesian procedure, ridge regression - Gaussian prior for *β*_*m*,*s*_ to avoid overfitting	- inferred motif activity profiles *β*_*m*,*s*_with set of TFs and miRNAs binding to sites of these motifs (= key regulators) - predicted target promoters, associated transcripts and genes - Network of known interactions between predicted targets and predicted regulatory interactions - enriched ontology categories
biRte	- mRNA differential expression - miRNA, TF measurements, CNV (optionally) - regulator (*R*) – target network	Likelihood model: ${L}_{D,\theta}(R)= p\left( D\Big| R,\theta \right)={\displaystyle \prod_{\widehat{D}} p}\left(\widehat{D}\Big| R,\theta \right) = {\displaystyle \prod_{\widehat{D}}{\displaystyle \prod_c{\displaystyle \prod_i p\left({\widehat{D}}_{i c}\Big|{R}_c,\theta \right)}}}$	- data specific marginal likelihoods using estimation of hidden state variables with via MCMC - Nested effects model structure Learning to reconstruct transcriptional network	- Estimation of active regulators - Estimation of associated transcriptional network
ARACNE	- microarray expression profiles	none	- local estimation of pairwise gene expression profile mutual information	- Reconstruction of gene regulatory network

Gene expression data is named “g” with index i, estimated parameters with “β”, TF binding information with “b”, TFs with “t”, samples with “s”, miRNAs with “mi” and model constants with “c”. Other variables are explained in the text

### Mathematical framework

**Fig. 2:**
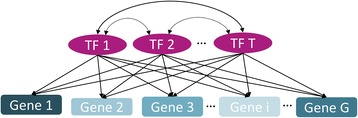
General scheme of a TF – gene network where all *T* TFs are connected to each other and can regulate all of the *G* genes

To combine regulatory networks and quantitative omics data and to thereby deduce regulatory activity, all methods described here use a genome-wide mathematical model. Sample specific gene expression values *g*_*i*,*s*_, derived from one biological condition, i.e., grouped into a single class, for in total *G* genes and *S* samples need to be provided as input. The background regulatory network is represented as a directed graph where the nodes designate regulators and regulated entities (mostly TFs and genes, but also miRNAs, regulatory sites, or TF complexes) and directed edges indicate a regulatory relationship between the two connected nodes, for example the influence of a TF on the expression of a gene (see Fig. 2).

We will use the variable *t* for regulators, *i* for regulated entities, and *b*_*t*,*i*_ for the strength of an edge from a TF/miRNA *t* to a gene *i* representing, for instance, a binding affinity. As abstract framework for explaining the different methods we propose a simple linear model predicting gene expression $\widehat{g_{i, s}}$ of gene *i* in sample *s* in terms of the activity of all *T* transcription factors *β*_*t*,*s*_, which regulate *i*, and the binding affinities *b*_*t*,*i*_. In contrast to Fig. 2, where TFs can influence each other, this model ignores TF – TF relations and feedback loops:

$$ \widehat{g_{i, s}}={\displaystyle \sum_{t=1}^T}{\beta}_{t, s}{b}_{t, i} $$

Given this model and a set of quantitative measurements of gene expression *g*_*i*,*s*_, the goal of the mathematical optimization is to find parameters *β* such that the sum of squared errors of measured vs predicted gene expression over all genes and samples is minimized using a certain norm, for example the L_2_ norm:

$$ \min {\displaystyle \sum_{i=1}^G}{\displaystyle \sum_{s=1}^S}{\left({g}_{i, s}-\widehat{g_{i, s}}\right)}^2 $$

### *Estimation of TF activity by the effect on their target genes* [31]

**Fig. 3:**
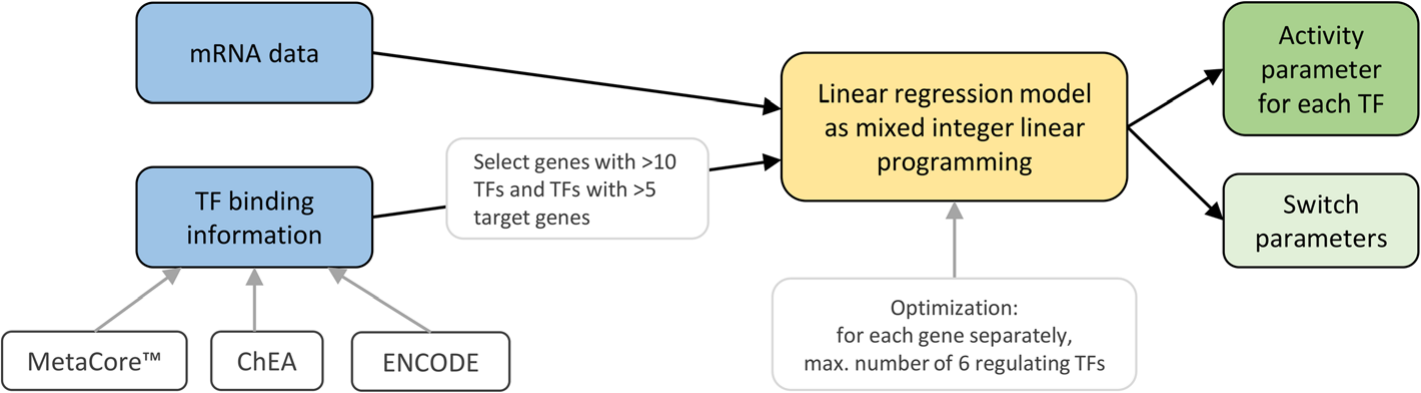
Flow chart of the approach by Schacht et al. The input data sets (marked in *blue*) are partly filtered and passed to a linear regression model (*yellow*) which calculates an activity value for each TF (*green*)

The idea of this method is to use the expression levels of TF’s target genes to infer their integrated effect (see Fig. 3). The method uses expression data and database curated TF binding information as input whereby the TF – gene network is restricted to genes regulated by more than 10 TFs and TFs with at least 5 target genes. The model is closely related to the abovementioned general framework, only adding a term for the sample specific effect of a TF. Specifically, the activity of a TF is modelled linearly by its cumulative effect on its target genes normalized by the sum of target genes or the TF’s gene expression level:

$$ \widehat{g_{i, s}}= c+{\displaystyle \sum_t}{\beta}_t{b}_{t, i}\left({\theta}_{a, t} ac{t}_{t, s}+{\theta}_{g, t}{g}_{t, s}\right) $$

where $\widehat{g_{i, s}}$ denotes the predicted gene expression of gene *i* in sample *s*, *c* is an additive offset, *β*_*t*_ describes the estimated activity of TF *t* and *b*_*t*,*i*_ refers to the underlying strength of the relation between TF *t* and gene *i* reflecting the binding affinity. The estimated effect of a TF in a certain sample is calculated via the switch-like term in parentheses, where either the activity definition $a c{t}_{t, s}=\frac{{\displaystyle {\sum}_i}{b}_{t, i}{g}_{i, s}}{{\displaystyle {\sum}_i}{b}_{t, i}}$ or the gene expression of the TF itself *g*_*t*,*s*_ is taken into account using the restrictions *θ*_*a*,*t*_, *θ*_*g*,*t*_ ∈ {0, 1} and *θ*_*a*,*t*_ + *θ*_*g*,*t*_ = 1. This switch term represents a meta-parameter to find the best model and has no biological interpretation. The model outputs an activity value and the information which switch parameter is chosen for each TF of the reduced network.

During the optimization, the sum of error terms (absolute value of the difference between predicted and measured gene expression) is minimized which is achieved via mixed-integer linear programming using the Gurobi 5.5 optimizer.^1^ The authors of this method state that the activity definition (see above) was used in 95% of their test cases, but the switch-like combination of both terms yielded still better optimization results. In the paper, the optimization task is greatly simplified as the model is computed for each gene separately and allows only a maximum number of 6 regulating TFs. The TF – gene network indicating the strength of a relation between a TF and a gene is created for 1120 TFs using knowledge from the commercial MetaCore™ database,^2^ ChEA [36] and ENCODE [15]. Due to the restriction of the network mentioned above, the actual model is then based on 521 TFs and 636 target genes only.

Evaluation of the results was performed using expression data from 59 cell lines of the NCI-60 panel [37, 38] and from melanoma cell lines (“Mannheim cohort”) [39]. A sample based leave-one-out and 10-fold cross validation of predicted and measured gene expression yielded Pearson correlation scores of about 0.6 for both data sets. A gene set enrichment analysis of the target genes for TFs modelled by the activity definition yielded 64 significantly enriched concepts including cell cycle, immune response and cell growth for the data from the NCI-60 panel. Additionally, a *t*-test was computed between melanoma and other cell lines of the NCI-60 panel to find differentially expressed genes of melanogenesis. For the resulting genes, regulation models were built and used to predict gene expression in the melanoma cell line data set yielding good prediction performances.

### *RACER* [32]

**Fig. 4:**
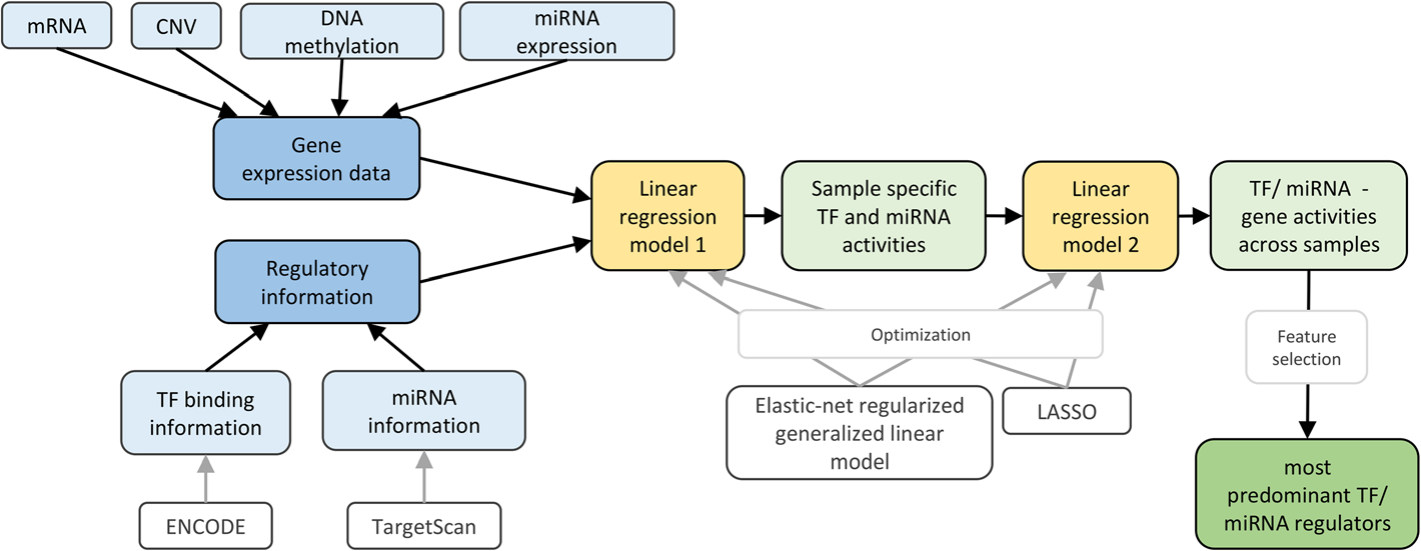
Scheme of RACER method. The input data sets (marked in *blue*) are passed to a two-step linear regression model (*yellow*) which calculates sample specific activity values for each regulator and determines the most predominant regulators (*green*)

RACER (Regression Analysis of Combined Expression Regulation) aims to integrate generic cell-line data with sample-specific measurements using a two-stage regression (see Fig. 4). Firstly, sample-specific regulatory activities for TFs and miRNAs are calculated. Subsequently, general TF/miRNA – gene interactions are derived.

Compared to our general framework, RACER includes additionally miRNA binding information. It assumes a linear combination, which is not further justified, of the regulatory effects of TFs and miRNAs on mRNA level. RACER can incorporate a variety of sample specific data including mRNA and miRNA expression values, CNV and DNA methylation. Optimization is applied twice to reduce model complexity, where the method first infers sample-specific TF and miRNA activities and uses these, in a second step, to compute general TF/miRNA – gene interactions.

In the first regression step, mRNA, miRNA, CNV and DNA methylation data are used to calculate the sample specific activities:

$$ \widehat{g_{i, s}}= c+{\theta}_{CNV, s} C N{V}_{i, s}+{\theta}_{DM, s} D{M}_{i, s}+{\displaystyle \sum_{t\ }}{\beta}_{t, s}\ {b}_{t, i} + {\displaystyle \sum_{mi\ }}{\beta}_{mi, s}\ {c}_{i, mi} miRN{A}_{mi, s} $$

where $\widehat{g_{i, s}}$ denotes the predicted gene expression of gene *i* in sample *s*, *c* is an intercept, *β*_*t*,*s*_ describes the estimated activity of TF *t* in sample *s* and *b*_*t*,*i*_ is the TF – gene binding score for TF *t* and gene *i*. The parameter *β*_*mi*,*s*_ stands for the estimated activity of miRNA *mi* in sample *s* and is multiplied by *c*_*i*,*mi*_, the number of conserved target sites on 3’UTR of the target gene *i* for miRNA *mi*, and by the expression level of miRNA *mi* in sample *s. θ*_*CNV*,*s*_ (respectively *θ*_*DM*,*s*_) are the regression parameters for CNV signals *CNV*_*i*,*s*_ (respectively DNA methylation data *DM*_*i*,*s*_). Using *β*_*t*,*s*_ and *β*_*mi*,*s*_ from the first regression step, TF – gene and miRNA – gene interactions across all samples are calculated in a second model:

$$ \widehat{g_{i, s}} = \tilde{c}+{\tilde{\theta}}_{i, CNV} C N{V}_{i, s}+{\tilde{\theta}}_{i, DM} D{M}_{i, s}+{\displaystyle \sum_{t\ }}{\gamma}_{i, t}\ {\beta}_{t, s} + {\displaystyle \sum_{mi\ }}{\gamma}_{i, mi}\ {\beta}_{mi, s} $$

where the sums apply only to a number of selected TFs and miRNAs with nonzero binding signals *b*_*t*,*i*_ > 0 and conserved target sites *c*_*i*,*mi*_ > 0. The resulting parameters *γ*_*i*,*t*_ and *γ*_*i*,*mi*_ indicate the strength of a TF/miRNA – gene relationship across all samples. To obtain robust estimates, *γ*_*i*,*mi*_ is additionally weighted by the averaged activities of the miRNA.

In each of the two regression steps, the optimization criterion is to minimize the sum of squared errors with L_1_ penalty on the linear coefficients to induce a sparse solution and to set irrelevant parameters to zero after the fitting. This sparse LASSO solution is obtained through elastic-net regularized generalized linear models. A supplementary feature selection procedure comparing the full model to a restricted model leaving one TF or miRNA out provides the most predominant TF/miRNA regulators. TF binding scores are collected from the generic cell line of erythroleukemia cells K562 from ENCODE for 97 TFs and 16653 genes. Further, the number of conserved target sites on 3’UTR is taken from sequence-based information from TargetScan for 470 miRNAs and 16653 genes. The RACER method is implemented in R and publicly available under http://www.cs.utoronto.ca/~yueli/racer.html.

The method was evaluated using expression data from an acute myeloid leukemia (AML) data set from TCGA with 173 samples [40] via a sample based 10-fold cross validation on the prediction of gene expression. To assess the quality of predictions, the Spearman rank correlation was calculated resulting in a reassuring value of approximately 0.6. Further, the full model was compared to models excluding one type of the input variables. The full model performed best and a substantial reduction of Spearman correlation was observed by omitting TF regulation (20%) and DNA methylation (5%). RACER also performed with competitive accuracy in predicting known miRNA – mRNA and TF – gene relationships compared to other methods like GenMiR++ [41] or ENCODE TF binding scores [15] using e.g., validated interactions from the MirTarBase [42] and knockdown studies. The feature selection procedure revealed 18 predominant transcriptional regulators in the AML dataset. Using their associated targets, a functional enrichment analysis showed that DNA repair and the tumor necrosis factor pathway were enriched. When applying this panel to cluster patients at different cytogenetic risks, the clustering pattern of the regulatory activities was largely consistent with the risk groups. Further, a literature survey on AML showed that many TF regulators among the top predictions had a role in leukemogenesis.

### *RABIT* [33]

**Fig. 5:**
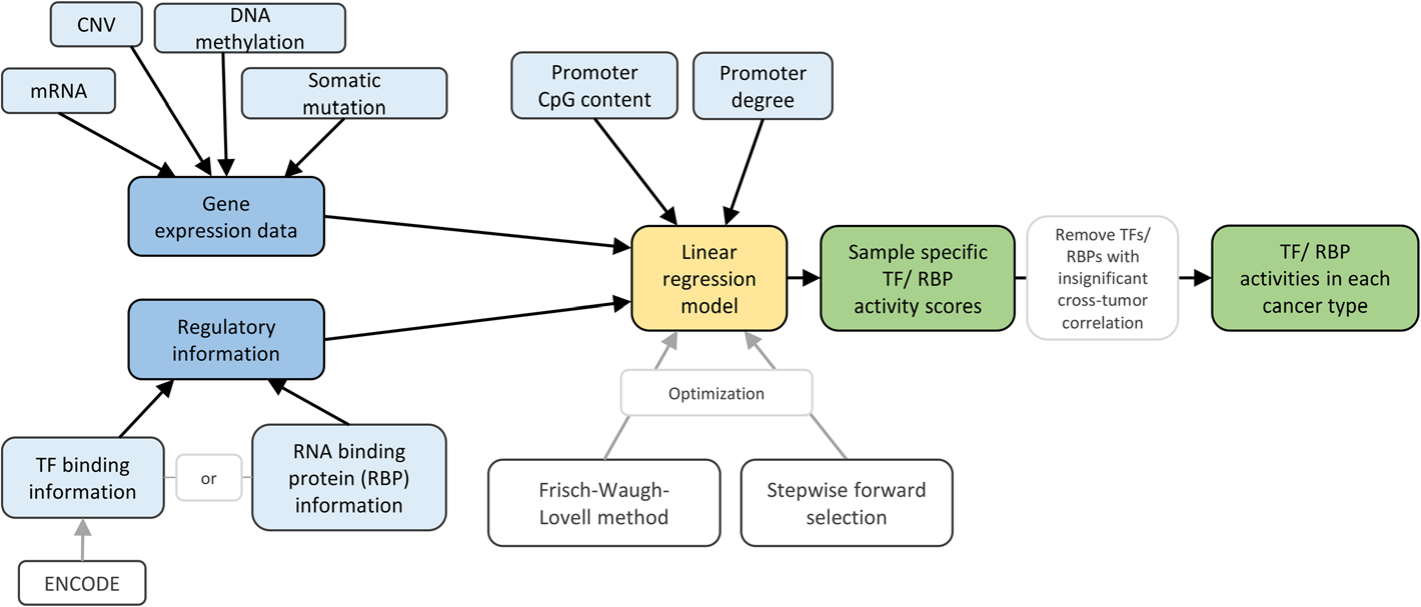
Flow chart of RABIT method. The input data sets (marked in *blue*) are passed to a linear regression model (*yellow*) which calculates sample specific activity values for each regulator and determines general regulatory activities (*green*)

Regression Analysis with Background Integration (RABIT) is a method for finding expression regulators in cancer by a large scale analysis across diverse cancer types. It integrates TF binding information with tumor profiling data to search for TFs driving tumor-specific gene expression patterns (see Fig. 5). It can be applied to predict cancer-associated RNA-binding protein (RBP) recognition motifs which are key components in the determination of miRNA function [43].

In contrast to our general framework, RABIT can, like RACER, make use of CNV and DNA methylation data additionally integrating promoter CpG content and promoter degree information (total number of ChIP-seq peaks near the gene transcription start site) and takes RBP or TF binding information as regulatory input. The computational model consists of three steps (see Fig. 5). First, RABIT tests in each tumor whether the target genes, identified by the BETA method [44], show differential expression compared to the normal controls including a control for background effects from CNVs, promoter DNA methylation, promoter CpG content and promoter degree:

$$ \widehat{g_i} = {\displaystyle \sum_f}{\theta}_f{B}_{f, i} + {\displaystyle \sum_t}{\beta}_t{b}_{t, i} $$

where $\widehat{g_i}$ represents the predicted differential gene expression between tumor and normal samples in gene *i*, *B* includes values of the *f* different background factors for gene *i*, *b* contains RBP or TF binding information and *θ* and *β* are the respective regression parameter vectors. The regression coefficients *β* are estimated by minimizing the squared difference between measured and predicted gene expression. The regulatory activity score for each TF/RBP is defined by a t-value (regression coefficient divided by standard error) and its significance by the corresponding *t*-test. If multiple profiles exist for the same TF from different conditions or cell lines, the profile with the highest absolute value of TF regulatory activity score is selected. In a second step, a stepwise forward selection is applied to find a subset of TFs among those screened in step one optimizing the model error. Lastly, TFs with insignificant cross-tumor correlation are removed from the results.

Computationally, the regression coefficients are calculated via the efficient Frisch-Waugh-Lovell method. TF binding information is taken from 686 TF ChIP-seq profiles from ENCODE representing 150 TFs and 90 cell types. Additionally, recognition motifs for 133 RBPs and their putative targets are collected by searching recognition motifs over the 3’UTR regions [45]. An implementation of the RABIT method can be downloaded from http://rabit.dfci.harvard.edu/download.

RABIT was applied to 7484 tumor profiles of 18 cancer types from TCGA using gene expression, somatic mutation, CNV and DNA methylation data. To systematically assess the results, the cancer relevance level of a TF was calculated as percentage of tumors with the TF target genes differentially regulated (averaged across all TCGA cancer types). A comparison to cancer gene databases, i.e., the NCI cancer gene index project [46], the Bushman Laboratory cancer driver gene list [47, 48], the COSMIC somatic mutation catalog [49] and the CCGD mouse cancer driver genes [50], showed a consistent picture. Further, RABIT’s performance was compared to other regression models like LAR or LASSO where RABIT had the best classification results when classifying all TFs into three categories by NCI cancer index and achieved better cross-validation error and shorter running time. The regulatory activity of RBPs showed that some alternative splicing factors could affect tumor-specific gene expression by binding to target gene 3’UTR regions.

### *ISMARA* [34]

**Fig. 6:**
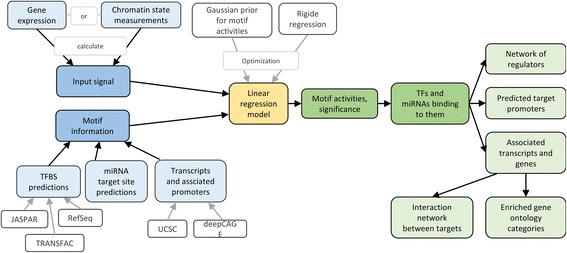
ISMARA model scheme. The input data sets (marked in *blue*) are passed to a linear regression model (*yellow*) which calculates motif activities and determines associated regulators (*green*)

In contrast to the previous three methods and to our general framework which directly scores TFs or other regulators, ISMARA (Integrated System for Motif Activity Response Analysis) infers the activity of regulatory motifs (short nucleotide sequences) and thereby indirectly deduces the effects of TFs and miRNAs (see Fig. 6). ISMARA is a web service where no parameter settings or specific processing of the input data, gene expression or ChIP-seq data are necessary. It can also be used to calculate regulatory activity differences between samples and consider replicates or data from time series.

ISMARA takes sample specific measurements and information about regulatory motifs for TFs and miRNAs into account. Based on the input of gene expression data or chromatin state measurements, the input signal is calculated for each promoter in each sample. The input signal levels are modelled linearly in terms of the binding site predictions and unknown motif activities:

$$ \widehat{g_{p, s}}={c}_p+{c}_s+{\displaystyle \sum_m}{N}_{p, m}\ {\beta}_{m, s} $$

where $\widehat{g_{p, s}}$ refers to the input signal for a promoter *p* in sample *s*, *c*_*p*_ *and c*_*s*_ are intercepts for each promoter and sample, *N*_*p*,*m*_ summarizes the TF/miRNA binding site predictions (sum of the posterior probabilities of all predicted TF/miRNA binding sites for motif m in promoter p) and *β*_*m*,*s*_ stands for the estimated motif activities. Like in the other presented methods, the optimization criterion is to minimize the sum of squared error terms between predicted and measured gene expression. Primarily, ISMARA provides the inferred motif activity profiles (*β*_*m*,*s*_) sorted by significance and a set of TFs and miRNAs that bind to these motifs representing the key regulators. Further, a list containing their predicted target promoters, associated transcripts and genes, a network of known interaction between these targets and a list of enriched gene ontology categories is displayed. The web service ISMARA is available under http://ismara.unibas.ch.

ISMARA employs a Bayesian procedure with a Gaussian likelihood model and a Gaussian prior distribution for *β*_*m*,*s*_to avoid overfitting. Information about regulatory motifs is provided via the annotation of promoters based on deep sequencing data of transcription start sites. To obtain a set of promoters and their associated transcripts, the 5’ ends of mRNA mappings from UCSC genome database are clustered with the promoters. TF binding site predictions in the proximal promoter region are collected using 190 position weight matrices representing 350 TFs from JASPAR, TRANSFAC, motifs from the literature and their own analyses of ChIP-seq and ChIP-chip data. Additionally, miRNA target sites for about 100 seed families are annotated in the 3’UTRs of transcripts associated with each promoter.

For evaluation, ISMARA was applied to data from well-studied systems and results were compared to the literature. Inferred motif activities were highly reproducible and even more robust than the expression profiles from which motif activities were derived. When comparing samples from 16 human cell types (GEO accession number GSE30611) from younger and older donors, ISMARA was able to identify a key regulator of aging-related changes in expression of lysosomal genes. A joint analysis of the human GNF atlas of 79 tissues and cell lines [51] and the NCI-60 reference cancer cell lines [52] revealed that many of the top dysregulated motifs were well-known in cancer biology like HIF1A and has-miR-205 miRNA. They also suggested novel predictions for regulating TFs in innate immunity, mucociliary differentiation and cancer.

### *biRte* [35]

**Fig. 7:**
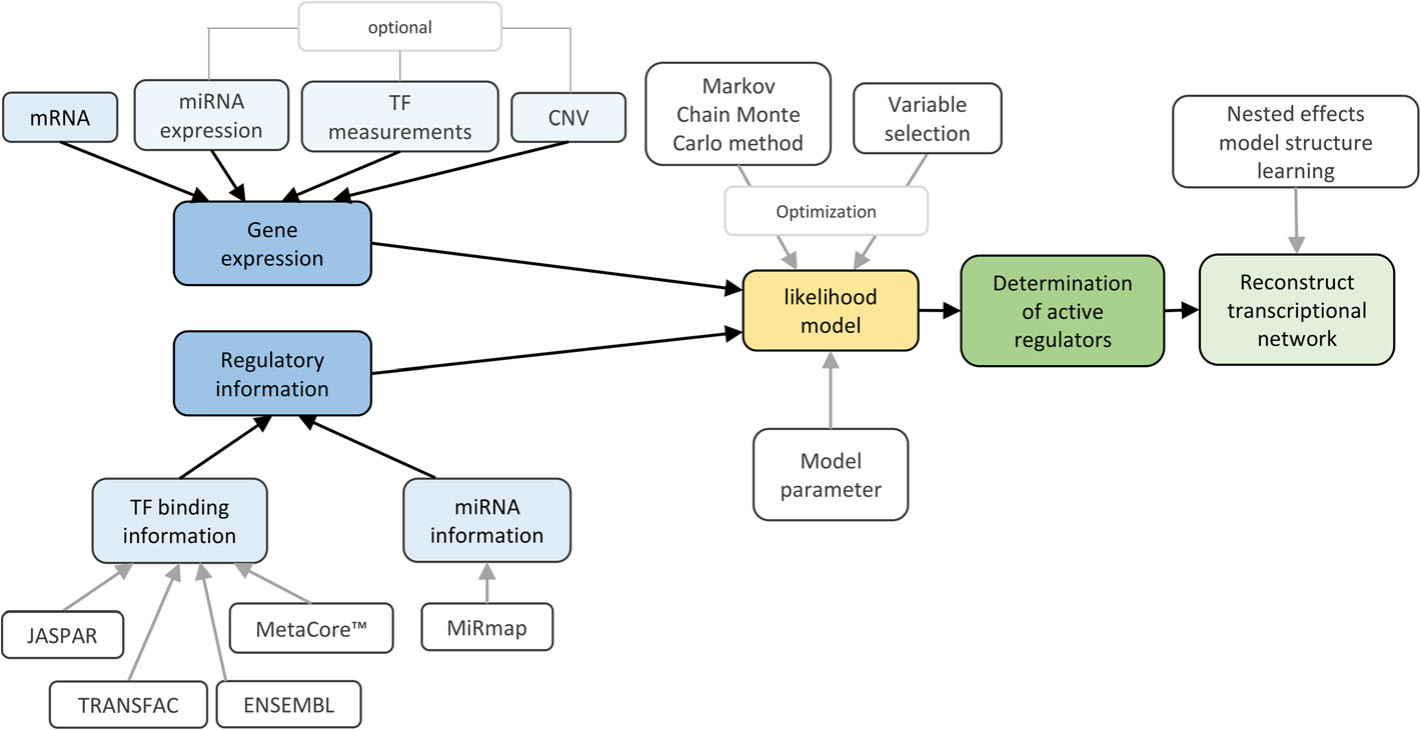
Scheme of biRte method. The input data sets (marked in *blue*) are passed to a likelihood model (*yellow*) which determines active regulators (*green*)

BiRte (Bayesian inference of context-specific regulator activities and transcriptional networks) takes a mathematically different approach compared to the abovementioned methods integrating TF/miRNA target gene predictions with sample specific expression data into a joint probabilistic framework (see Fig. 7). Compared to our general scheme of a TF – gene network ( Fig. 2), biRte takes the TF/miRNA – gene network without the interactions between regulators to estimate regulatory activities and infers the network between regulators in a second step.

BiRte takes as input differential gene expression data (mRNA), an underlying regulatory network including TF/miRNA – target gene binding information and optionally CNV data, miRNA and TF expression measurements. As opposed to our general framework, biRte defines a likelihood model for the set of active TFs/miRNAs (called regulators *R* which can be seen as hidden variables) based on the entire gene expression data *D* and certain model parameters *θ*:

$$ {L}_{D,\theta}(R)= p\left( D\Big| R,\theta \right)={\displaystyle \prod_{\widehat{D}} p\left(\widehat{D}\Big| R,\theta \right)} = {\displaystyle \prod_{\widehat{D}}{\displaystyle \prod_c{\displaystyle \prod_i p\left({\widehat{D}}_{i c}\Big|{R}_c,\theta \right)}}} $$

Here *D* represents the set of all available experimental data including mRNA, CNV, miRNA and TF expression data and *D*_*ic*_ refers to its *i*th feature measured under experimental condition *c*. The condition specific hidden state variables *R*_*c*_ are estimated with help of the Markov Chain Monte Carlo (MCMC) method where a regulator can switch from an active to an inactive state (switch) or an inactive and an active regulator exchange their activity states (swap). Thereby, the posterior probability for each regulator and condition to influence the expression of its target genes is estimated. Simultaneously, a variable selection procedure is applied to achieve sparsity of the model. The optimization goal is not, as one would expect, to return the configuration with highest posterior probability among all sampled ones but to take marginal selection frequencies during sampling into account and filter those above a defined cutoff. After the determination of active regulators, the associated transcriptional network containing TFs and miRNAs is inferred from the observable differential expression of target genes and target gene predictions for individual regulators.

In practice, the stochastic sampling scheme based on MCMC allows swap operations only when regulators show a significant overlap of regulated targets. The variable selection procedure is implemented via a spike and slab prior [53] which can integrate prior knowledge about the activity of regulators. To infer the associated transcriptional network, Nested Effects Model (NEM) [54] structure learning is applied. An input miRNA – gene network is constructed based on MiRmap [55] for 356 miRNAs. The TF – target gene network with 344 TFs is compiled by computing TF binding affinities to promoter sequences according to the TRAP model [56] using data from ENSEMBL, TRANSFAC, JASPAR and MetaCore™. An implementation of biRte is available for R on Bioconductor under https://bioconductor.org/packages/release/bioc/html/birte.html.

Several simulations were conducted to study model behavior. On the basis of a human regulatory sub-network and accordingly simulated expression data of 900 target genes biRte was compared to BIRTA [57], GEMULA [58] and a hypergeometric test and further to other network reconstruction algorithms like ARACNE [30], GENIE3 [59] and GeneNet [60]. BiRte performed best in regulator activity predictions including a favorable computation time and was robust against false positive and false negative target gene predictions. Additionally, biRte was applied to an E.coli growth control and to a prostate cancer data set including 44 normal and 47 cancer samples from GEO (GSE29079) with corresponding array data from 464 human miRNAs (GSE54516) and the results showed a principal agreement with the biological literature.

### ARACNE [30]

**Fig. 8:**
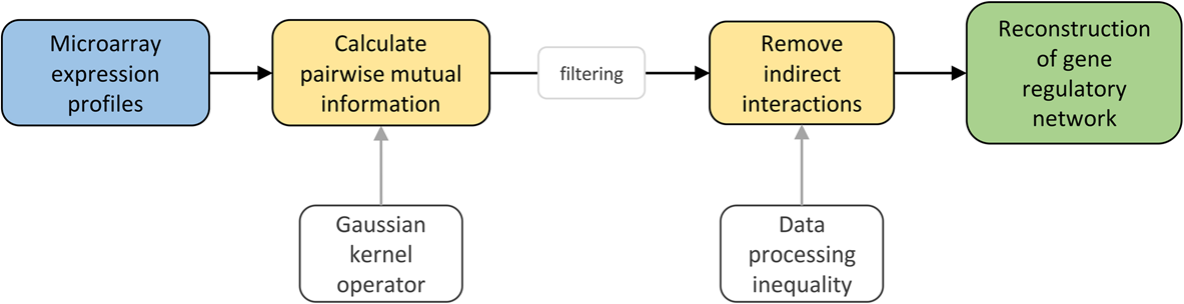
ARACNE flow chart. The input data set (marked in *blue*) is used to calculate pairwise mutual information where indirect interactions are removed (*yellow*) and which allow a reconstruction of the gene regulatory network (*green*)

We compare ARACNE (Algorithm for the Reconstruction of Accurate Cellular Networks) [30] as an established, yet local, tool for the reconstruction of gene regulatory networks to the previous five recent genome-wide approaches. The algorithm is background knowledge-free and identifies transcriptional interactions based on mutual information including non-linear and non-monotonic relationships and distinguishes between direct and indirect relationships (see Fig. 8). ARACNE is a free tool available under http://califano.c2b2.columbia.edu/aracne.

ARACNE uses as input only microarray expression profiles and estimates candidate interactions by calculating the pairwise gene expression profile mutual information *I* defined as

$$ I\left({g}_i,{g}_j\right)={I}_{i, j}= S\left({g}_i\right)+ S\left({g}_j\right)- S\left({g}_i,{g}_j\right) $$

where S denotes the entropy. *I*_*i*,*j*_ measures the relatedness of genes *g*_*i*_ and *g*_*j*_ and equals zero if both are independent. In a second step, the mutual information values are filtered using an appropriate threshold depending on the distribution of all mutual information values between random permutations of the original data set and indirect interactions are removed.

Computationally, a Gaussian kernel operator is used to calculate mutual information scores. In a subsequent step, the data processing inequality (DPI) [61] is applied to remove probably indirect candidate interactions. The DPI states that if the genes *g*_*i*_ and *g*_*k*_ interact only through a third gene *g*_*j*_, then

$$ I\left({g}_i,{g}_k\right)\le \min \left( I\left({g}_i,{g}_j\right), I\left({g}_j,{g}_k\right)\right) $$

Thus, the least of the three mutual information scores can come from indirect interactions only [30].

ARACNE’s performance was evaluated on the reconstruction of realistic synthetic datasets [62] and on an expression profile dataset consisting of about 340 B lymphocytes derived from normal, tumor-related and experimentally manipulated populations [63] against Relevance Networks and Bayesian networks. Regarding the synthetic networks, ARACNE had consistently better precision and recall values compared to the two other algorithms and reached very good precision at significant recall levels. It recovers far more true connections and fewer false connections than the other methods with better performance on tree-like topologies compared to scale-free topologies. A reconstructed B-cell specific regulatory network was found to be highly enriched in known c-MYC targets where about 50% of the predicted genes to be first neighbors were reported in the literature.

## Results

We described five recent methods for the genome-wide inference of regulatory activity, namely the approach by Schacht et al., RACER, RABIT, ISMARA, and biRte. They all assume the topology of the regulatory network to be known, cast activity estimation as an optimization problem regarding the difference between predicted and measured values, take different types of sample specific omics data into account, and eventually produce a list of regulators like transcription factors or miRNAs, ranked by their estimated activities in the samples under study. We also included ARACNE which is background knowledge-free and uses only local dependency measures to reconstruct a regulatory network and indirectly infer activities. All of the presented methods essentially follow the same goal, i.e., accurate ranking of regulatory activity, but differ in the types of measurements being integrated, the background knowledge necessary for their application, the complexity and refinement of the underlying model of gene regulation, and the concrete paradigm used for solving the optimization problem. Most of the methods, except for the approach by Schacht et al., are available online via a downloadable implementation, a web service, or an R package providing an operable solution for the interested user. Whereas an overview of the main features of each method ca be found in Table 1, we now first compare the algorithms regarding their general properties in a descriptive way.

The data sets used for evaluation vary between all methods. Therefore, we further implemented an evaluation framework to compare the method by Schacht et al., RACER, RABIT and biRte in an objective and quantitative way. We used experimental data of three publicly available data sets from TCGA [64] and a regulatory network as background knowledge. We first used only mRNA expression data as input to the four methods to ensure the result’s comparability, whereas in a second evaluation step, also other omics data sets were included where possible. We further analyzed the relevance of regulators found by different methods using a literature search.

### General properties

#### Experimental data types included

The methods differ in the types of measurements being integrated, which corresponds to the level of detail of their model of gene regulations. All six methods use mRNA as input. RACER, RABIT and biRte can also integrate CNV, DNA methylation, TF/miRNA expression data, or somatic mutations. ISMARA calculates an input signal from microarray, RNA-seq, or ChIP-seq data.

Additionally, all presented methods use prior knowledge about the underlying regulatory network. These networks are extracted from different data sources and pre-processed in different manners. All methods require at least knowledge about TF – gene relationships, yet RACER, biRte and ISMARA also incorporate information about miRNAs. When using RABIT, the user can choose whether to provide TF or RNA binding protein information. The approach of Schacht et al. and biRte extract regulatory information partly from the commercial MetaCore™ database, whereas the other methods use only publicly available databases, like ENCODE, JASPAR or TRANSFAC. The networks which are used for the evaluations published in the respective papers are publicly available for the case of RACER (network for 16653 genes, 97 TFs and 470 miRNAs), RABIT (predicted binding scores of 63 RBP motifs and 17463 genes) and biRte (network for E.coli including 160 TFs). Neither Schacht et al. nor ISMARA make this data available.

#### Mathematical models of regulatory activity

The methods use different mathematical models to infer regulatory activity. The approach by Schacht et al., RACER, RABIT and ISMARA use linear regression whereas biRte applies a probabilistic framework. ARACNE, as a local method, is based on mutual information. RACER and RABIT can be seen as extensions of the approach by Schacht et al. since they essentially use the same model structure but incorporate more input data types and more classes of regulatory information. Further, RACER applies a two-stage regression to infer regulatory activity.

#### Optimization frameworks

For assessing regulator activities, Schacht et al., RACER, RABIT and ISMARA minimize the sum of error terms between measured and predicted gene expression. However, the methods use rather different algorithms for solving the resulting optimization problem, and also apply different constraints to achieve model sparsity, robustness of inference, and feature selection. In the approach by Schacht et al., the regression model is computed for each gene separately and allows only a maximum number of six regulating TFs. RACER uses a LASSO approach, while ISMARA follows a Bayesian model that infers regulator activities as posterior distributions. LASSO can be interpreted as a Bayesian model using Laplacian priors instead of Gaussian priors in the regression framework obtaining point estimates of the regulatory activities and enforcing sparseness of the solution [32]. In contrast, biRte uses a likelihood model with a spike and slab prior to induce model sparsity. This approach implements a selective shrinkage of model coefficients such that estimates are less biased compared to a LASSO prior [65]. With the help of the spike and slab prior, sparsity can be controlled in a variable dependent manner allowing the inclusion of prior belief in the activity of each regulator [35].

#### Computed outputs

Schacht et al. and biRte determine activity of regulators over all samples at once, whereas RACER and biRte first infer sample-specific activities which are combined to cross-tumor activities only in a second optimization step. In contrast, ISMARA in first place infers motifs activity; these activities are used to deduce the effects of TFs and miRNAs by their motif binding profiles. ISMARA primarily provides sample specific TF and miRNA activity but also offers an option to group samples and compare average regulatory activity between different conditions. Like biRte and ARACNE, it also infers the network of the regulators themselves.

#### Methods and data sets used for evaluation

The type and extent of evaluation performed for the different methods vary greatly. They range from direct application to biological problems over the comparison of results to the biological literature to simulation studies. All methods published evaluations results on publicly available datasets, e.g., from the National Cancer Institute, TCGA or GEO, but unfortunately address different tissues and cancer types. Sample-based cross-validation is applied in the work by Schacht et al., RACER, RABIT and ISMARA. The first two of these methods use correlation coefficients between measured and predicted gene expression for assessing prediction quality. RACER, RABIT and biRte compare their results to the outcome of other algorithms and to those of restricted models, for example excluding one type of the input variables. All methods search the literature to compare their predictions to previously published studies on the respective biological question. Overall, ISMARA provide the most extensive biological evaluation using a battery of relevant use cases, whereas biRte excels in systematic simulation studies. Sadly, there are very few works which compare any of the methods presented on the same problem; the only result we are aware of compared ARACNE and biRte regarding their performance in network reconstruction on simulated data, in which biRte attained higher robustness against false positive and false negative target gene predictions [35].

### Quantitative comparison

Although certain evaluation steps were carried out for all methods, results in the original papers are not comparable as they used different input datasets, different background regulatory networks, and different evaluation metrics. Therefore, in addition to the comparison of general properties of the methods, we implemented an evaluation framework using three independent and publicly available test data sets to compare the method by Schacht et al., RACER, RABIT and biRte in an objective and quantitative way. All evaluated methods were given the same regulatory network as input.

#### Data sets

For the evaluation we used experimental data from TCGA [64] for three cancer types: Colon adenocarcinoma (COAD), liver hepatocellular carcinoma (LIHC) and pancreatic adenocarcinoma (PAAD). For all three cancer types, mRNA expression, CNV, DNA methylation and miRNA expression data is available for primary tumor and normal tissue samples. These data sets are openly accessible via the NCI Genomic Data Commons Data Portal^3^ or the NCI Genomic Data Commons Legacy Archive^4^ (DNA methylation data).

For mRNA gene expression we used processed RNA-seq data in the form of FPKM (fragment per kilobase of exon per million mapped reads) values. The files included Ensembl Gene IDs which were converted to HGNC symbols using the Ensembl [66] BioMart tool^5^ to match the IDs of the TF – gene network. In two cases, when multiple Ensembl Gene IDs mapped to one HGNC symbol, we chose the gene with highest log2 fold change between case and control group. miRNA expression was given as RPM (reads per million miRNA mapped) measurements. Both mRNA and miRNA data were centered using a weighted mean such that the mean of the case group equaled the negative mean of the control group, and normalized via a weighted standard deviation. CNV data was retrieved as masked copy number segment where the Y chromosome and probe sets with frequent germline copy-number variation had already been removed. Chromosomal regions were mapped to genes using the R package biomaRt [67]. If multiple records mapped to one gene, the median of the segment mean values was calculated. For DNA Methylation data we used the beta-values of Illumina Human Methylation 450 arrays as methylation scores. Multiple scores for the same gene were averaged within a sample.

We restricted our analyses to the samples for which all four input data types were available. When multiple measurements for one sample and data type were available, we used only the first one in alphabetical order of the file name. After this selection procedure, 165 samples remained for COAD, 404 for LIHC and 180 for PAAD. A list including sample and file information is available in Additional file 1.

Together with the experimental data, all evaluated methods were given the same regulatory network as input. We used a publicly available human TF – gene network [28] based on a text-mining approach and complemented it with TF – gene interactions from the public TRANSFAC^6^ database [19]. This network included 2894 interactions between 429 TFs and 1218 genes. The network is provided in Additional file 2.

#### Evaluated methods

We conducted the quantitative comparison for the method proposed by Schacht et al., RACER, RABIT and biRte. ISMARA was not included since it is (a) only available as a web service, (b) can only be used with its own, proprietary underlying regulatory network model, and (c) requires the upload of raw data which is prohibited by TCGA’s terms of use. Also ARACNE [30] was not included in the quantitative evaluation since it does not use background knowledge and we therefore consider its results as incomparable to the other methods.

- For the approach by Schacht et al. we re-implemented their method as closely as possible to the original design using Python and the Cuneiform workflow language [68, 69]. Due to the high number of integer parameters in the original method, the complexity of optimizing the whole network at once would have by far exceeded computational measures. Therefore, like in the original paper, we computed the model for each gene separately and restricted the number of regulating TFs per gene to six. We added a second step where we used these TF – gene interactions building a sub-network to optimize TF activity globally to describe the interplay of the TFs’ effects on their target genes. As in the implementation of Schacht et al., we used the Gurobi Optimizer.^7^
- For RACER we used the available R scripts^8^ and extracted the resulting sample-specific regulatory activities.
- RABIT published a C++ implementation which they provide on their website^9^ and which we used with the FDR option set to 1. As RABIT takes differential expression into account, we used the difference of expression values between case and control group as input and ordered the TFs by t-value as proposed in the RABIT paper.
- BiRte is available as a bioconductor R package. We used R version 3.3.2 with biRte version 1.10.0 and applied the method “birteLimma” to estimate regulatory activities with the options niter and nburnin set to 10000. As biRte has a randomized component, the resulting TF activities are not exactly the same for different runs. We averaged the final activity scores over 1000 iterations of birteLimma.

For our re-implemented method by Schacht et al. and RACER we computed separate models for case and control group and ranked the TFs by their activity difference between the two groups.

To ensure the result’s comparability, we first used only mRNA expression data as input to the four methods. In a second evaluation, we included also other omics data sets where possible. BiRte was evaluated on mRNA and CNV data, RABIT on mRNA, CNV and DNA methylation data, and RACER additionally used miRNA expression as input. We obtained lists with the regulators ranked according to the absolute value of their computed activity for each cancer type and method, with and without the use of additional inputs. For each cancer type we calculated the size of the overlaps in the four different results using the top 10 and top 100 regulators. The results for the top 10 regulators using either only mRNA or multiple omics data sets as input are shown in Table 2.

**Table 2:**
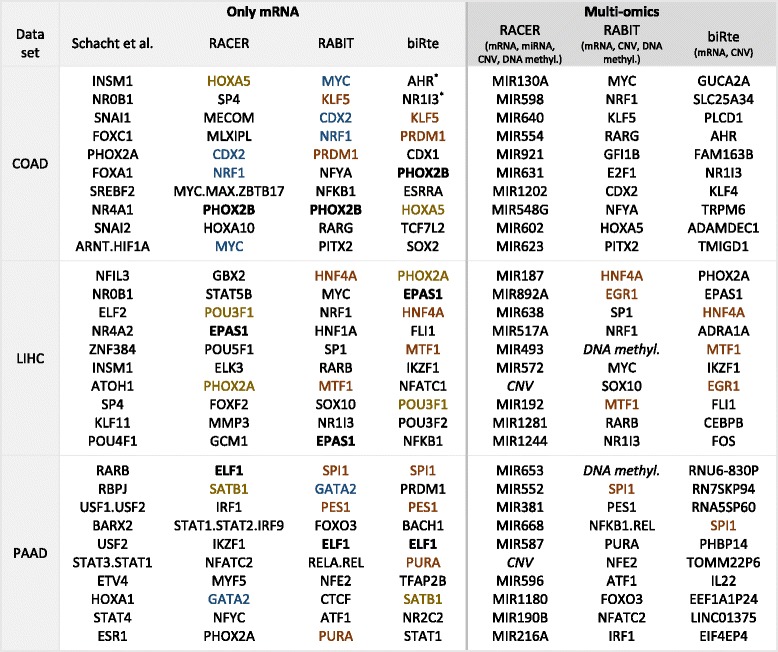
HGNC Symbols of the top 10 regulators found by each method for COAD (using 165 samples), LIHC (404 samples) and PAAD (180 samples) and the use of only mRNA data as input (left panel) and multiple input data sets (RACER: mRNA, miRNA, CNV and DNA methylation; RABIT: mRNA, CNV and DNA methylation; biRte: mRNA and CNV; right panel). TFs with equal activity values are marked with*. TFs found by several method’s top 10 are marked in bold (when found by RACER, RABIT and biRte), blue (RACER and RABIT), red (RABIT and biRte) or yellow (RACER and biRte)

#### Only mRNA as input

When only mRNA is used as input, one TF is commonly found by the three methods RACER, RABIT and biRte in each data set, respectively: PHOX2B for COAD, EPAS1 for LIHC and ELF1 for PAAD. A literature search of these TFs and their targets revealed clear associations to the respective cancer type. The TF obtained commonly for COAD, PHOX2B, is related to TLX2, a gene which has been shown to play a role in the tumorigenesis of gastrointestinal stromal tumors [70]. EPAS1, which was found in the LIHC top 10 TFs of three methods, is linked to CXCL12, which plays an important role in metastasis formation of hepatocellular carcinoma by promoting the migration of tumor cells [71, 72]. For PAAD, three methods ranked TF ELF1 high, which is related to 14 genes in our network, inter alia to BRCA2 and LYN. Mutations in the BRCA2 gene have been implicated in pancreatic cancer susceptibility [73, 74], whereas the knockdown of LYN reduced human pancreatic cancer cell proliferation, migration, and invasion [75]. These results underline that the methods are able to find biologically relevant information about regulation processes in cancer.

Several TFs in the top 10 are found by two of the four methods For instance, RACER and RABIT have four common top 10 TFs (CDX2, NRF1 and MYC next to PHOX2B) in the COAD data set. However, the top 10 TFs found by the method by Schacht et al. do not overlap with any top 10 TFs of the other methods in any data set. The agreement of RACER, RABIT and biRte in the top 10 TFs hints to the biological importance of the found TFs since this overlap is statistical significant as the probability of finding common TFs in three sets of ten randomly chosen ones out of 429 TFs (*p*-value) is below 0.006. Additionally, the methods do identify different TFs for different data sets, indicating the importance of the actual cancer specific mRNA expression values and that results are not dictated by the background network.

**Fig. 9:**
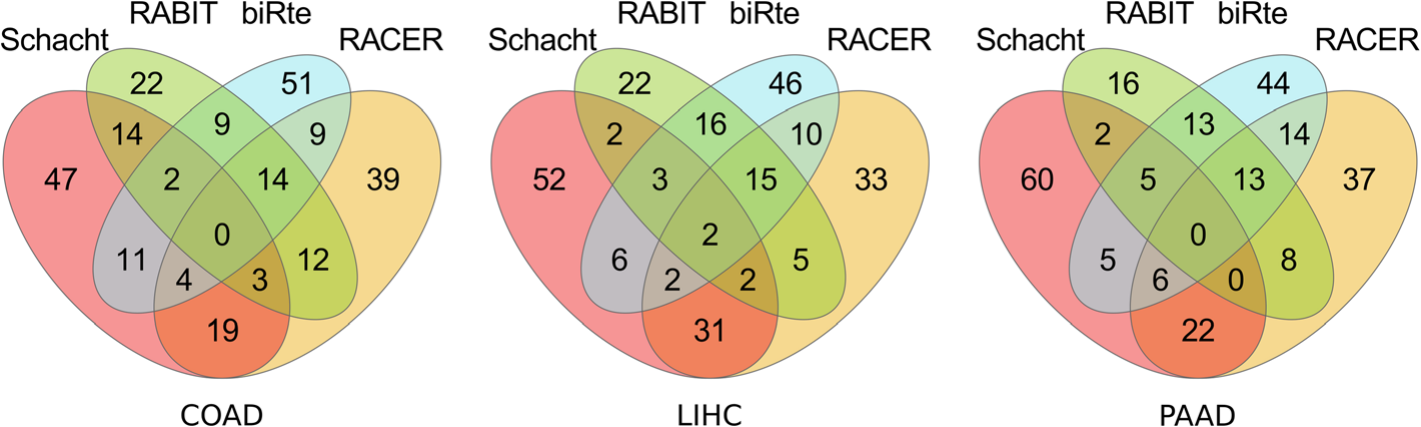
Number of overlapping TFs in the top 100 of ranked TFs per method (for RABIT the overlap with the top 76/67/57 TFs (having activity > 0) in COAD/LIHC/PAAD is shown)

The results for the number of overlapping regulators in the top 100 between the four methods and the three different data sets are shown in Fig. 9. For RABIT, only 76 TFs for COAD (resp. 67 for LIHC and 57 for PAAD) could be ranked since all other TFs had an activity value equal to zero.

When looking at the overlap of three of the four methods, the number of overlapping TFs is still the highest for the triplet RACER, RABIT and biRte. For the LIHC dataset two TFs are found in the top 100 of all four methods (E2F4 and SOX10). E2F4 is a downstream target of ZBTB7, which was associated to the expression of cell cycle-associated genes in liver cancer cells [76]. Two target genes of E2F4, CDK1 and TP73 were also involved in liver cancer development [77] and proposed as prognostic marker of poor patient survival prognosis in hepatocellular carcinoma [78]. Further, epigenetic alterations of the EDNRB gene, a target of SOX10, might play an important role in the pathogenesis of hepatocellular carcinoma [79]. Even if the result of four methods finding two common TFs is not statistically significant (*p*-value = 0.36), their association to liver hepatocellular carcinoma shows that the methods reach their goal of identifying relevant TFs.

However, when comparing different data sets, the methods tend to rank the same TFs under the top 100 to a greater or lesser extent. For example, the overlap of all top 100 TFs of the three cancer types is only one TF for RABIT and nine TFs for biRte, but 16 TFs for the method by Schacht et al. and even 32 TFs for RACER. Therefore, the results from RABIT and biRte seem to be more cancer type specific and less dependent on the regulatory network than the results from RACER. However, we did not specifically investigate the influence of the underlying network and its topology on the results which would be an interesting point for further research.

#### Multi-omics data as input

When not only taking mRNA into account but also miRNA, CNV and DNA methylation, the results are more difficult to compare between the methods, since they all use a different way of combining different types of data due to their models and implementations.

We are aware of the lower level of comparability of this approach regarding the multi-omics results in contrast to a scenario, where all methods are evaluated on the same set of input data. However, we intended to use maximum set of input data for each method to cover the effect of the use of multiple omics data sets compared to only mRNA as input.

BiRte was evaluated on mRNA and CNV data, RABIT on mRNA, CNV and DNA methylation data, and RACER additionally used miRNA expression as input. Whereas RACER and RABIT considered CNV or DNA methylation data as one background factor and compute only one activity value, biRte evaluated the influence of each CNV separately.

The results (see Table 2, right panel) show that RACER exclusively ranks miRNAs high; not a single TF is found among the top 10 regulators. Also, the influence of CNVs was high in LIHC and PAAD. However, the TFs that RACER found in the top 10 when using only mRNA data as input are still ranked high in the multi-omics scenario, e. g the COAD top three TFs of the mRNA results are ranked 13th, 16th and 14th in the results of the multi-omics input. The difference of the results coming from the two input types is less for RABIT: seven TFs are still in the top 10 for COAD (8 for LIHC and 6 for PAAD) when using CNV and DNA methylation additionally to mRNA data. Therefore, the contribution of additional input data seems not to be crucial for the performance of RABIT. BiRte considers each CNV as a potential regulator which increases the total number of regulators enormously. Still, two commonly present TFs in the top 10 of the COAD data set (even six for LIHC and one for PAAD) are found by either the sole mRNA input and the multi-omics approach.

The overlap of the top 10 of RABIT and biRte in the multi omics case is considerable with three TFs in LIHC (HNF4A, EGR1 and MTF1; *p*-value = 0.001), and one TF in PAAD (SPI1; *p*-value = 0.21). Three of them (HNF4A, MTF1 and SPI1) were already found when using only mRNA data as input.

The results for the use of different input data sets show that the top ranked regulators are drastically changed when using additionally miRNA data in RACER, but change less when only CNV or DNA methylation data is provided in RABIT and biRte. However, the results from multi omics analyses are difficult to compare since the combination of input data sets is not consistent across the three different methods.

## Discussion

### Background networks

A crucial input to the models is the underlying regulatory network which is needed to reduce the search space for actual regulatory activity. However, the construction of comprehensive TF/miRNA – gene regulatory networks is difficult for various reasons. Firstly, a comprehensive characterization of the human regulatory repertoire is lacking since only about half of the estimated 1,500–2,000 TFs in the mammalian genome is known [80]. ChIP experiments, prone to a high false positive rate [81], were used to identify TF binding patterns but each assay is limited to the detection of one TF in one condition and therefore TF binding has not been characterized for many TFs in most cell types. Further, the local proximity of a binding site to the transcriptional start site of a gene does not automatically implicate transcriptional regulation. With regard to posttranscriptional regulators, the functions for only a few of the around 1,200 different miRNAs have been experimentally determined and current data on miRNA targets is mostly based on computational predictions [82]. Generally, the knowledge about TF and miRNA binding is scattered over the biological literature and different, partly commercial, databases, impeding the construction of comprehensive networks [28]. Therefore, any comparative evaluation of the methods presented here would have to make sure that the same background network is used for each computation. Besides, studies on the impact of network incompleteness or different error rates in networks would be important to assess the ability of the methods to cope with such common problems. Simulation studies will be vital in this regard.

### The graph view on regulation

The modelling of regulatory networks as graphs, as used in all presented methods, is perhaps not the optimal representation for the underlying biological regulatory processes. A graph cannot easily account for important effects such as TF complex formation and temporal and spatial synchronization of activities. Furthermore, TF binding is affected by chromatin state and the impact of posttranslational modifications on transcriptional activity which are difficult to include in a graph view on regulation. The model’s dependence on the topological structure and the robustness to changes in the underlying network have not been evaluated or discussed in any of the presented methods even if these issues are known to have a severe influence in network analysis [83].

### Underlying mathematical model

Linear models, widely spread in different fields of science, provide a simple and easily understandable design but over-simplify the underlying biological processes. Nonlinear behavior, e.g., saturation effects, cannot be represented. Considering that the number of available samples is typically relatively small, the incorporation of many different data types and according parameters into the model could result in excessively complex designs prone to overfitting, but this issue lacks general awareness. Only two of the presented methods incorporate parameter priors (ISMARA and biRte), and two apply cross validation techniques to estimate prediction performance (method by Schacht et al. and RACER). Further, the effect of temporal buffering between TF binding and the actual effect on gene expression is not included in any of the methods.

### Comparability

All methods produce a ranked list of regulators. Comparing these results across different methods, even when applied on the same data set and using the same background network, is difficult since no generally accepted benchmarks are available. Therefore, there currently is no objective measure to designate a best method. The closest comparable evaluation effort we are aware of is implemented in the “DREAM5 – Network Inference” challenge [84], which targets gene regulatory network reconstruction. The invited participants reverse-engineered a network from gene expression data, including a simulated network, and evaluated the results on a subset of known interactions or the known network for the in-silico case. The approach of GENIE3 [59] which trains a random forest to predict target gene expression performed best and the integration of predictions from multiple inference methods showed robust and high performance across diverse data sets. However, an extensive competitive evaluation to determine active regulators based on a given regulatory network has, to the best of our knowledge not been carried out yet.

We therefore compared the results of four methods in a quantitative way. The experimental data and the regulatory network we used as input are publicly available to ensure transparency of our results. The results suggest that the methods are able to find biologically relevant information about regulation processes in cancer. However, the result overlaps are rather low (though sometimes statistically significant). This seems surprising as all methods essentially follow the same goal, i.e., identification of the most differentially active TFs or genes. We think further research is necessary to exactly characterize the specific strengths of each method. Furthermore, we did not investigate the influence of the underlying network on the results, which is another topic for further research.

## Conclusion

Despite their often rather involved procedures and models, none of the presented methods adequately reflects the biological reality of regulatory activity in cells. A specific disease phenotype is rarely caused by a single gene but rather a product of the interplay of genetic variability, epigenetic modifications and post-transcriptional regulation mechanisms [85]. The presented methods ignore a multitude of such factors like the effects of chromatin state and alternative splicing, nonlinear relationships between regulatory activity and gene expression, or kinetic and temporal effects. Furthermore, TFs themselves regulate the expression of other TFs forming feedback loops which are not considered in any of the presented methods. Nevertheless, the methods apparently are able to detect strong signals and produced promising results in terms of ranking transcription factors by their activity and are thus valuable tools for identifying biomarkers for specific phenotypes.

## Additional files

Additional file 1: Lists information about the samples and files from TCGA included in our quantitative evaluation for all three cancer types (COAD, LIHC and PAAD). (XLS 697 kb)

Additional file 2: Includes an adjacency list of the connected nodes of the TF – gene network. The list includes three columns (“TF”, “gene”, “edge”) where each row indicates an association with the value of “edge” between a TF and a gene. Complexes of TFs are indicated with a separating “.” between their components. (TXT 39 kb)

## Footnotes

1
http://www.gurobi.com/products/gurobi-optimizer

2
http://lsresearch.thomsonreuters.com/pages/solutions/1/metacore

3
https://gdc-portal.nci.nih.gov

4
https://gdc-portal.nci.nih.gov/legacy-archive

5
http://www.ensembl.org/biomart/martview, release 87

6
http://www.gene-regulation.com/pub/databases.html, release 7.0

7 version 6.04, available under a free academic license

8
http://www.cs.utoronto.ca/~yueli/racer.html (accessed 17 October 2016)

9
http://rabit.dfci.harvard.edu (accessed 05 February 2016)
